# The role of the blood–brain barrier during neurological disease and infection

**DOI:** 10.1042/BST20220830

**Published:** 2023-03-17

**Authors:** Adjanie Patabendige, Damir Janigro

**Affiliations:** 1 Brain Barriers Research Group, Department of Biology, Edge Hill University, Ormskirk, U.K.; 2 Department of Clinical Infection, Microbiology and Immunology, University of Liverpool, Liverpool, U.K.; 3 The School of Biomedical Sciences and Pharmacy, The University of Newcastle, Newcastle, NSW, Australia; 4 Flocel Inc., Cleveland, OH, U.S.A.; 5 Department of Physiology and Biophysics, Case Western Reserve University, Cleveland, OH, U.S.A.

**Keywords:** Blood–brain barrier, cerebral ischaemia, flow disturbances, inflammation, neurological disorders, SARS-CoV-2

## Abstract

A healthy brain is protected by the blood–brain barrier (BBB), which is formed by the endothelial cells that line brain capillaries. The BBB plays an extremely important role in supporting normal neuronal function by maintaining the homeostasis of the brain microenvironment and restricting pathogen and toxin entry to the brain. Dysfunction of this highly complex and regulated structure can be life threatening. BBB dysfunction is implicated in many neurological diseases such as stroke, Alzheimer's disease, multiple sclerosis, and brain infections. Among other mechanisms, inflammation and/or flow disturbances are major causes of BBB dysfunction in neurological infections and diseases. In particular, in ischaemic stroke, both inflammation and flow disturbances contribute to BBB disruption, leading to devastating consequences. While a transient or minor disruption to the barrier function could be tolerated, chronic or a total breach of the barrier can result in irreversible brain damage. It is worth noting that timing and extent of BBB disruption play an important role in the process of any repair of brain damage and treatment strategies. This review evaluates and summarises some of the latest research on the role of the BBB during neurological disease and infection with a focus on the effects of inflammation and flow disturbances on the BBB. The BBB's crucial role in protecting the brain is also the bottleneck in central nervous system drug development. Therefore, innovative strategies to carry therapeutics across the BBB and novel models to screen drugs, and to study the complex, overlapping mechanisms of BBB disruption are urgently needed.

## Introduction

The blood–brain barrier (BBB) formed by brain capillary endothelial cells is the dynamic physiological structure that protects the brain to maintain normal neuronal function. These highly specialised endothelial cells have intercellular tight junctions such as claudins, occludin, zonula occludens, and junctional adhesion molecules that control the movement of molecules through the paracellular pathway (‘gate function’) by showing size and charge selectivity. An array of specific transporters, receptors and enzymes controls the molecular traffic via the transcellular route and permit the passage of nutrients and removal of waste products across the BBB. Tight junctions also act as a ‘fence’ to segregate these transporters to the apical and basal domains, so that the endothelium can act as a polarised barrier to prevent free movement of the transporters. The presence of complex intercellular tight junctions results in high transendothelial electrical resistance (TEER) in brain microvessels, compared with peripheral microvessels. In addition, brain endothelial cells lack fenestrations, have very few pinocytotic vesicles, and reduced expression of adhesion molecules, which limit immune cell infiltration (for a comprehensive review on the BBB see [1]).

Disruption of the BBB can leave the brain vulnerable to damage. Understanding the detailed cellular and molecular mechanisms of BBB disruption has been a long-standing interest in the field. However, in many neuropathological conditions, whether BBB dysfunction is a causative or consequence of the pathology remains incompletely understood. Investigating these mechanisms can be challenging, due to the complexities with direct assessment of BBB function. However, recent developments in the field have provided some insights. This review aims to evaluate some of these latest developments and summarises the findings with a particular focus on the effects of inflammation and flow disturbances on the BBB during neurological disease and infection, and discusses whether timing of BBB disruption plays a role.

**Figure 1.**
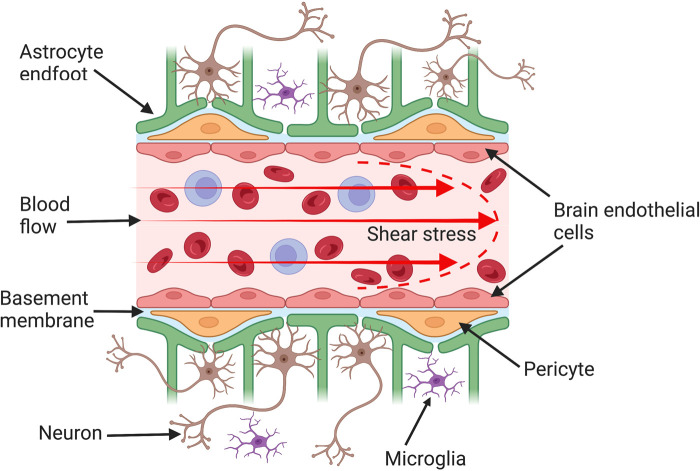
Structure of the blood–brain barrier (BBB). The BBB is formed by brain endothelial cells that line cerebral capillaries and is the main protective physiological barrier preventing the entry of toxins and pathogens into the brain. These endothelial cells are supported by other cells such as astrocytes and pericytes of the neurovascular unit, which are important in inducing and maintaining BBB characteristics. Brain endothelial cells differ from those of peripheral tissues by having more complex intercellular tight junctions that restrict the paracellular permeation of molecules through the junctional cleft. Furthermore, endothelial cells *in vivo* are continuously exposed to shear stress (the frictional force generated by blood flow), which affects endothelial cell structure and function.

An intact BBB is extremely important for regulating central nervous system (CNS) homeostasis to maintain normal neuronal function, and to protect the brain from fluctuations in plasma neurotransmitter levels, circulating pathogens and toxins. However, the BBB does not function independently, but is in continuous ‘cross-talk’ with other cells [2] such as astrocytes [3,4], pericytes [5,6], neurones [7,8], perivascular macrophages [9], microglia [10] and immune cells [11]. The unique relationship between these cells and the BBB gave rise to the concept of the neurovascular unit (NVU) (Figure 1).

## Role of astrocytes and pericytes in supporting the BBB

The NVU is a relatively new concept. However, the critical importance of astrocytes in the induction and maintenance of BBB structure and function has long been established (reviewed in [12]). Brain capillaries are surrounded by perivascular endfeet of astrocytes and therefore astrocytes occupy a strategic position between brain capillaries and neurons. This close proximity and the ability of astrocytes to secrete soluble factors [13,14] allow them to induce BBB phenotype in brain endothelial cells. Most *in vitro* BBB models use astrocytes in co-culture to take advantage of the astrocyte-secreted factors to increase the tightness/TEER of brain endothelial cells [15,16]. These *in vitro* studies have provided a great deal of information to support the role of astrocytes in up-regulating many BBB features, including low paracellular permeability and up-regulation of tight junctions [14,17,18], transporters [19–22] and enzymes [23]. Although astrocytes are important for the development and maintenance of the BBB, recent evidence suggests that it is pericytes that are critical for the formation and induction of the BBB and are implicated in contributing to the progression of CNS disease.

Pericytes are embedded in the basement membrane in the abluminal surface of brain microvessels. Unlike astrocytes, they only partially cover the microvessel, but their processes can span several endothelial cells. Due to a lack of pericyte-specific markers, pericytes can be difficult to differentiate from other mesenchymal cells such as vascular smooth muscle cells that also sit in the basement membrane [24]. Pericytes extend their processes along and around pre-capillary arterioles, capillaries and post-capillary venules, and may have different morphological and functional features depending on their position along the vascular tree [25]. The heterogeneity of pericyte morphology and the lack of specific pericyte markers have led to controversy and confusion about their functions [26,27]. Nevertheless, pericytes have been shown to have diverse functions including regulating BBB permeability and integrity [6,28], cerebral blood flow regulation and neurovascular coupling [29,30], secreting neuroinflammatory mediators [31], involvement in fibrosis following neuronal injury [32], cognitive decline and Alzheimer's disease [33,34] clearance of neurotoxins [35], providing neurotrophic support [36], maintaining white matter structure and function [37], potential to transform into multipotent stem cells [38,39] and are critically important for angiogenesis [40,41] and induction of the BBB.

Convincing evidence for the role of pericytes in BBB induction came from studies using mice that were genetically modified to have defects in pericyte generation by targeting the platelet-derived growth factor (PDGF)-B/PDGF receptor-β (PDGFR-β) signalling pathway. These studies showed that many BBB properties such as expression of occludin, claudin-5, zonula occludens-1 and BBB influx transporter Glut-1 were present a week earlier than astrogliogenesis [5], at the time of pericyte recruitment by endothelial cells via PDGF-B secretion during early embryogenesis [42]. Pericyte recruitment coincided with BBB sealing, as the pericyte-deficient mice had increased BBB permeability and increased expression of leukocyte adhesion molecules. They showed that the extent of the pericyte coverage determined BBB permeability. Remarkably, the basis for this increased permeability was an up-regulation of endothelial transcytosis and not a lack of tight junctions [5,6]. Thus, pericytes are critical during BBB development, and maintain BBB properties by inhibiting the expression of ‘leaky’ BBB features (such as transcytosis and expression of leukocyte adhesion molecules) to stabilise vessels, as they do not directly affect tight junction protein expression. Interestingly, this study also showed that pericytes express cues for guiding astrocyte endfeet attachment to endothelial cells, and the absence of pericytes led to abnormal endfeet polarisation [6]. Therefore, pericytes seem to play an important role in orchestrating the proper formation of the BBB and the NVU. Given that astrocytes are unlikely to be required for BBB induction, they are expected to be important for maintaining the fully differentiated BBB phenotype during adulthood and involved in BBB response to disease.

## Mechanisms of BBB disruption

**Figure 2.**
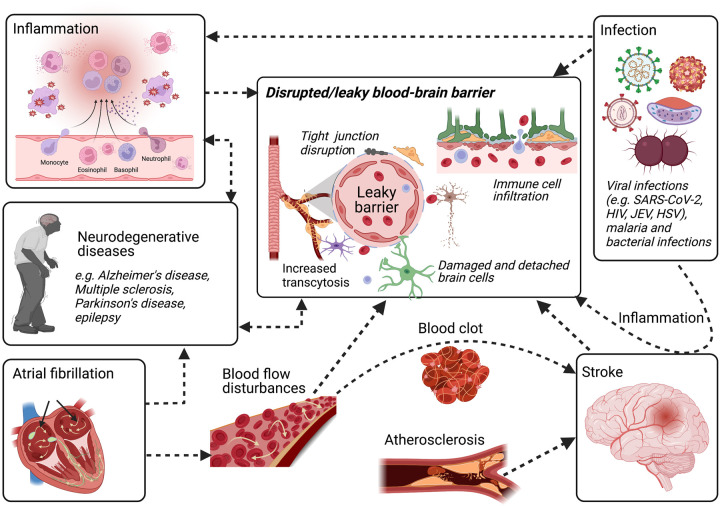
Blood–brain barrier (BBB) disruption during neurological infections and diseases. BBB disruption is implicated in many neurological infections and diseases, including neurodegenerative diseases, ischaemic stroke as well as viral infections such as SARS-CoV-2, and peripheral diseases such as atrial fibrillation. Disruption of the BBB may involve the opening of tight junctions, damage to the endothelium, increased transport of molecules across the BBB (transcytosis) and alterations in transport systems. Inflammation is a final common pathway in many neurological diseases, allowing immune cell and pathogen entry to the brain, which leaves the brain vulnerable to damage. Furthermore, abnormal flow patterns or flow cessation can lead to changes in shear stress or pulsatility. This can deteriorate the brain endothelium and lead to barrier impairment. Deterioration in neuroprotective BBB function plays a major role in the pathogenesis of disease since the BBB dynamically responds to many events associated with flow disturbances, oxidative stress and proinflammatory cytokine generation. Any condition that affects the functional integrity of the BBB will cause secondary effects on cerebral blood flow and vascular tone, resulting in further damage to the brain.

Destabilisation of the BBB occurs under or leads to several pathological conditions including stroke [43,44], Alzheimer's disease [45], multiple sclerosis [46,47], epilepsy [48–50], viral encephalitis [51–53], COVID-19 [54–56], Neuro Aids [57], malaria [58–60] and sequelae of traumatic brain injury [61] as well as peripheral diseases such as atrial fibrillation (AF) [62] (Figure 2). While some of the mechanisms of BBB disruption are CNS-derived (e.g. microglia, glutamate), others are peripheral in origin. Dysfunction of the BBB during most neurological diseases can lead to increased permeability (due to disrupted tight junctions and increased transcytosis) [63–65], leading to immune cell infiltration, [46] neuroinflammation [66] and oedema [67,68]. Leukocytes, interleukins, and other soluble factors are known to disrupt the integrity of the BBB [69–71]. Therefore, anti-inflammatory manoeuvres could prevent or repair the BBB [72–74]. In addition, disruption in ion regulation and transporter function in BBB [43,75–77] has also been reported, which severely impact neuronal function [78]. The initiation of this damage can be either due to a direct assault on the BBB, for example during infections, or due to secondary neurological damage, which leads to activation of neuroinflammatory pathways as is the case of ischaemic stroke.

Increased BBB permeability leading to cerebral oedema [68,79] and haemorrhagic transformation [80,81] are common complications of ischaemic stroke, which can impact the outcome of these patients with potential serious and life-threatening consequences [82]. A series of factors or events in combination or in sequence can lead to BBB disruption in ischaemic stroke. For example, several factors including reactive oxygen species (ROS) [83], pro-inflammatory cytokines [67,84] and matrix metalloproteinases (MMPs) [85–88] have been implicated in BBB damage after stroke. Neuronal injury following ischaemia leads to the release of proinflammatory mediators such as tumour necrosis factor-alpha (TNF-α), interleukin-6 (IL-6), IL-1α, IL-1β and interferon-γ (IFN-γ) that can activate microglia, astrocytes and endothelial cells, promoting the expression of molecules that can contribute to NVU breakdown [83,89]. This local inflammation leads to the up-regulation of adhesion molecules such as vascular cell adhesion molecule-1 (VCAM-1), intercellular adhesion molecule-1 (ICAM-1), P-selectin in endothelial cells and secretion of MMPs such as MMP2 and MMP9 [86,88]. The enhanced expression of adhesion molecules in brain endothelial cells attracts leukocytes and platelets, and promotes the tethering/rolling, firm adhesion and transmigration of these cells across the BBB [90]. Adhesion of leukocytes causes further damage due to the activation of signalling pathways and release of ROS and inflammatory cytokines and MMPs from other cells of the NVU. This creates a proinflammatory environment, which further activates the endothelium and leads to increased BBB permeability. Increased permeability due to inflammation has been demonstrated using magnetic resonance imaging (MRI) at both the acute and chronic phases of stroke in patients with ischaemic stroke [91,92] and in animal models [93,94].

BBB damage due to inflammation is a final common pathway in many brain infections as well, allowing pathogens and immune cells access to the brain, and leading to CNS damage. For example, an inflammatory cytokine-mediated pathway is implicated for the BBB disruption seen in encephalitis caused by viruses such as Japanese encephalitis virus (JEV) [51,95], herpes simplex virus (HSV) [52,53], West Nile virus (WNV) [96,97] and human immunodeficiency virus (HIV) [98,99] and in severe acute respiratory syndrome coronavirus 2 (SARS-CoV-2) [56,100]. Several studies have provided evidence for an important role for inflammation in BBB disruption and disease outcome. In clinical samples from patients with Japanese encephalitis, increased levels of pro-inflammatory cytokines and chemokines in cerebrospinal fluid (CSF) were shown to be associated with poor outcome [101]. Another study using human autopsy material showed that perivascular inflammation and a damaged BBB are associated with widespread perivascular oedema [102]. Further studies using *in vitro* human BBB models have provided evidence for JEV-induced inflammation causing increased BBB permeability [51,103]. Pro-inflammatory cytokine milieu seems to be main the driving force contributing to increased BBB permeability in JEV [51]. This was also demonstrated in a mouse model where the virus was detected in the brain and the onset of inflammation occurred prior to BBB disruption [95]. It is now established that JEV does cross the BBB, causing neurological damage primarily due to the resulting inflammatory response.

Mechanisms of neuroinvasion of other viruses such as SARS-CoV-2 are still under investigation. SARS-CoV-2 has been detected in the brains of patients with severe disease who present with neurological symptoms [104–106]. Among others, the most common neurological symptoms presented in hospital by patients infected with SARS-CoV-2 are stroke, seizures and encephalitis or meningitis [107]. BBB disruption is generally implicated in these neurological manifestations. A post-mortem study of patients with COVID-19 demonstrated microvascular damage and fibrinogen leakage, indicating a disrupted BBB. However, the study failed to detect SARS-CoV-2 in the brain, possibly due to the virus being cleared at the time of death or detection limitations in the assay used [108]. Another study by the same authors showed a significant increase in serum protein leakage, platelet accumulation as well as increased expression of platelet endothelial cell adhesion molecule-1 (PECAM-1) and von Willebrand factor in patients that died from COVID-19 compared with control subjects [109]. Furthermore, macrophages and some CD8+ T cells infiltration were also detected, similar to other neurological diseases where a leaky BBB leads to immune cell infiltration. The results suggest that SARS-CoV-2 infection could cause BBB disruption via activation of the endothelium leading to increased BBB permeability. Indeed, a recent study in COVID-19 patients has demonstrated that patients with neurological complications had the highest levels of biomarkers associated with BBB disruption, such as MMP9 [110].

The angiotensin-converting enzyme 2 (ACE2) has been shown to be the host receptor responsible for binding SARS-CoV-2 [111]. ACE2 is present in brain vascular endothelial cells [112] and the mechanisms of SARS-CoV-2 infection have been implicated in brain endothelial dysfunction and BBB damage, which may explain the neurological manifestations compounded by the effects of the systemic inflammatory response [113]. Convincing evidence from clinical/post-mortem studies is still lacking to provide a definite answer to whether BBB disruption due to SARS-CoV-2 infection is due to a direct infection of endothelial cells and/or the resulting inflammation. Results from animal and *in vitro* studies have provided some clues. SARS-CoV-2 can be detected in the brain [114] and that the virus is able to infect brain endothelial cells [115] and cause BBB disruption [116]. In addition, SARS-CoV-2 spike protein has been shown to increase BBB permeability, trigger a pro-inflammatory response (increased expression of cell adhesion molecules) and up-regulate the expression of MMPs in 2D static and 3D microfluidic *in vitro* human BBB models [56]. SARS-CoV-2 spike proteins have also been shown to induce brain endothelial dysfunction [117]. Another study using K18-hACE2 transgenic mice and Syrian hamsters demonstrated that SARS-CoV-2 can cross the BBB with direct infection of brain microvascular endothelial cells in both *in vivo* and *in vitro* models [118]. Infection led to an increase in BBB permeability with disrupted basement membrane via MMP9 mediated pathway. However, alterations in tight junctions were not observed. In agreement with other studies, pro-inflammatory modulators were also up-regulated. Others have reported conflicting findings, where SARS-CoV-2 replication in human brain endothelial cells was shown to be weak, and infection of the cells did not affect BBB integrity or lead to an increase in inflammation despite inoculating the cells with a high virus load [119]. It is possible that BBB disruption in COVID-19 is due to inflammation triggered by circulating pro-inflammatory cytokines in response to the systemic disease. Furthermore, the resulting leaky BBB could increase neuroinvasion of the virus, which could further exacerbate inflammation via triggering pro-inflammatory responses of other cells of the NVU.

Finally, it is also worth noting that stroke and COVID-19 pathophysiology exhibit overlapping molecular mechanisms that contribute to BBB disruption. In particular, in both conditions, inflammation plays a major role in the pathophysiology of CNS disease via vascular dysfunction. While in COVID-19, peripheral inflammation seems to be the major trigger of BBB disruption, in stroke, the inflammatory response of the cells forming the NVU plays a major role. Astrocyte activation in ischaemic stroke has a major impact on brain damage and repair mechanisms [120]. Similarly, Sánchez and Rosenberg propose that astrocyte dysfunction may play a major role in contributing to stroke development in COVID-19 patients, and that further understanding of the molecular pathways could provide potential insights into new therapies to combat neurological dysfunction due to SARS-CoV-2 infection [121].

## Timing and extent of BBB disruption during neurological disease

BBB disruption can manifest as a transient event with minor consequences on normal brain function or lead on to a chronic or total breakdown of the barrier properties, resulting in significant and/or irreversible brain damage. In neurological diseases such as stroke [122–126], traumatic brain injury [127,128] and Alzheimer's disease [129], as well as in some neurological infections [130], BBB disruption has been demonstrated to be biphasic in nature. For example, experimental studies in ischaemic stroke have provided extensive evidence that demonstrates an early opening of the BBB within the first 4–6 h of a stroke is followed by a refractory period and then a late opening ∼48–72 h [131,132]. However, there is also evidence to suggest BBB recovery is not complete following the initial opening, and that BBB continues to be leaky [133,134]. Some studies have also shown further disruption to the BBB where BBB permeability is increased after 7 days or more post-reperfusion [135,136]. Conflicting data from these studies could be the result of using different stroke models and methods for detecting BBB disruption, but could also be due to the heterogenous nature of the mechanisms responsible for BBB disruption and stroke severity.

While BBB tight junction protein disassembly and reassembly have been shown to be responsible for BBB disruption following ischaemia [125], mainly via degradation of tight junction proteins by MMPs [137–140], other mechanisms such as an increase in the number of endothelial caveolae, up-regulation of endothelial transcytosis [141] and disruption of the glycocalyx [126] have also been suggested to play an important role depending on the phase of BBB disruption. For example, Knowland et al.’s study using a novel transgenic mouse strain with Claudin-5 labelled with eGFP demonstrated that tight junction disruption only appeared at the late phase, ∼48–58 h post-stroke, while the rate of transcytosis and the number of endothelial caveolae increased in the early phase at 6 h post-reperfusion [141]. Traditional view of BBB tight junction disassembly during the early phase of reperfusion has been questioned by several studies, which strongly suggest that transcellular permeability rather than paracellular permeability is increased during this phase, given that these studies demonstrate normal tight junction morphology despite elevated vascular permeability/transcytosis at the BBB [126,142–146]. Therefore, maintaining low rates of transcytosis appear to be important in maintaining barrier properties following stroke, and therapeutics that target up-regulated transcytosis, which precedes tight junction abnormalities will have significant potential in preventing BBB disruption in stroke.

There is now also strong evidence to demonstrate that BBB breakdown could be considered as an early biomarker of neurological disease. For example, in individuals with early cognitive impairment, brain capillary damage and BBB disruption can be seen independently of Alzheimer's Aβ and/or tau biomarker changes [147]. Another study has demonstrated that in carriers of the main susceptibility gene for Alzheimer's disease (E4 variant of apolipoprotein E), breakdown of the BBB contributes to cognitive decline independently of Alzheimer's disease pathology [33]. In addition, age-dependent early BBB breakdown has also been implicated in the hippocampus, which worsened with mild cognitive impairment [148]. In patients with early Alzheimer's disease, increased BBB permeability is associated with cognitive decline [149]. These studies suggest that compromised BBB is an early indication of developing cognitive deficits and dementia.

## Role of shear stress in BBB

One of the distinctive features of endothelial cell physiology is their exposure to pulsatile shear stress [150,151]. This is a most neglected aspect for *in vitro* modelling of endothelial cells, where the majority of systems used lack a physiological level of shear stress [152]. When exposed to shear stress to levels comparable to *in vivo*, endothelial cells respond by morphological, transcriptional, and functional levels. In addition, it is becoming increasing accepted that altered shear stress promotes pathological changes in vascular function. At the morphological level, it has been shown that endothelial cells align with flow [153–155]. This is likely due to calcium influx-mediated cytoskeletal rearrangement [151,156]. Atherosclerosis-prone regions of larger arteries have endothelial cells exposed to irregular, complex flow patterns with a non-physiological magnitude of shear stress (low levels and rapidly changing direction) [157]. After complete flow cessation, endothelial cells re-enter cell cycle and ‘pile up’ onto each other [158–160]. This is commonly observed in traditional, no-flow models *in vitro* [160]. Transcriptional changes in endothelial cells exposed to shear stress were first reported by the Janigro group [158,159]. The changes in mRNA levels were primarily related to cytoskeletal remodelling, abolishment of cell cycle (mitotic arrest and differentiation), glucose metabolism and nicotinamide adenine dinucleotide (NADH) production (shift of glycolytic efficiency towards reduction mechanism to counter oxidative stress), and transporter levels and membrane positioning [20,161]. From the functional viewpoint, the main effect of shear is the control of vascular tone by a mechanism mediated by calcium entry and nitric oxide (NO) production by endothelial nitric oxide synthase (eNOS) [162–164]. NO released by endothelial cells causes smooth muscle relaxation and increased blood flow to the organ.

## Pathophysiology of BBB due to flow disturbances

At the pathological level, turbulent flow (altered shear stress) or flow cessation/reperfusion have been shown to alter vascular function by impeding many of the physiological mechanisms listed above. In addition, low NO levels have a prothrombotic effect, triggering the formation of emboli and ultimately causing stroke. A paradigm of pathological changes due to altered flow is evident during AF. AF, the most common cardiac dysrhythmia, is associated with poor outcomes, including stroke [165–167]. The incidence of stroke attributable to AF increases from 1.5% at age 50–59 years to 23.5% at age 80–89 years [168]. Over 12 million people worldwide have a stroke yearly [169], of which at least 25% have been directly attributed to clinically diagnosed AF [170]. The adverse effects of AF are due to haemodynamic changes with multiple factors leading to a prothrombotic state [171]. AF also affects the BBB (For a comprehensive review on the effects of AF on BBB, see [62]) and elevates biomarkers of cerebral injury [172]. A link between AF, the BBB and cognitive impairment has been proposed [62]. NO has been recognised as a key component in the regulation of vascular tone and in mediating the prothrombotic state in AF [171]. NO production by endothelial cells is reduced in AF [173,174]; reduced NO affects vessel diameter and increases the probability of thrombus formation [175]. Reversal of AF to normal rhythm also reverses endothelial cell dysfunction [176]; AF-like pacing reduces NO production by endothelial cells in an animal model and *in vitro* [177]. Finally, wall shear stress, the frictional force of blood flow tangential to an artery lumen, has been demonstrated in multiple studies to influence aneurysm formation and risk of rupture [178,179].

If one looks more specifically at the brain vasculature and the BBB, most of the findings on systemic vessels hold true. Intriguingly, Aryal and Patabendige have suggested that AF may be causative of cognitive decline by a mechanism involving altered shear stress [62]. This was also suggested in seminal translational articles on aging and the vascular unit [180,181]. However, only sparse data [157,158] are available addressing BBB-specific changes triggered by altered shear stress, since most of the literature has focused on endothelial sequelae after embolic or cardiogenic stroke and reperfusion. Novel tools will be required to study the effects of altered vascular perfusion on NO production and TEER in cultured brain endothelial cells exposed to pulsatile flow (but see [164,182–184]). Ideally, to study AF on brain vasculature endothelial cells one should devise a system where shear stress can be modulated beyond the usual parameters of increasing or decreasing steady-state perfusion (and shear), flow cessation/reperfusion, and static conditions. In particular, a system where AF-derived abnormal heart rhythms to trigger perfusion events would be useful to study acute and chronic effects on endothelial cell cultures exposed to pathological levels of intermittent shear and turbulence.

## Concluding remarks

A healthy brain is protected by the BBB, and is extremely important for the normal functioning of neurons. However, during many neurological diseases and infections, this barrier is disrupted, leaving the brain vulnerable to further damage. Worldwide, neurological disorders remain the main cause of disability and the second leading cause of death [185]. Therefore, novel therapies are urgently required. However, given the highly selective nature of the BBB, drug penetration into the CNS has always been a major hurdle in developing treatments for neurological disorders [186]. To overcome this hurdle, several innovative approaches are currently being developed. Some of the novel techniques include, using receptor-mediated transcytosis to deliver nanoparticles [187], cell-penetrating peptide conjugated adeno-associated viruses (AAVs) to deliver novel gene therapies [188], organic cation transporters 1 and 2 (Oct1/Oct2) to deliver novel stroke therapeutics [189], intranasal delivery methods [190], cell therapies [191] and focused ultrasound (FUS) in conjunction with gas-filled microbubble contrast agents [192]. Finally, to make progress, the development of suitably robust and reliable *in vitro* BBB models for high throughput drug screening as well as models that can mimic the complex pathophysiology of BBB dysfunction to elucidate the underlying mechanisms are essential.

## Perspectives

- BBB disruption is implicated not only in neurological infections and diseases but also in peripheral diseases, leading to CNS damage. Yet, our understanding of the complex mechanisms underlying the pathophysiology of BBB dysfunction remains incomplete.
- Deterioration of neuroprotective BBB function plays a major role in the pathogenesis of disease since the BBB dynamically responds to many events associated with inflammation and flow disturbances, which can cause brain damage.
- Novel models that closely simulate the structure and function of the BBB are required to study the complex cellular and molecular pathways that are disrupted during neurological disease. Furthermore, innovative strategies are required to deliver therapeutic drugs across the BBB for CNS disease.

## Open Access

Open access for this article was enabled by the participation of University of Liverpool in an all-inclusive *Read & Publish* agreement with Portland Press and the Biochemical Society under a transformative agreement with JISC.

## Abbreviations

ACE2: angiotensin-converting enzyme 2
AF: atrial fibrillation
BBB: blood–brain barrier
CNS: central nervous system
CSF: cerebrospinal fluid
JEV: Japanese encephalitis virus
MMPs: matrix metalloproteinases
NO: nitric oxide
NVU: neurovascular unit
ROS: reactive oxygen species
SARS-CoV-2: severe acute respiratory syndrome coronavirus 2
TEER: transendothelial electrical resistance

## References

[1] Abbott, N.J., Patabendige, A.A., Dolman, D.E., Yusof, S.R. and Begley, D.J. (2010) Structure and function of the blood–brain barrier. Neurobiol. Dis. 37, 13–25 10.1016/j.nbd.2009.07.030 19664713

[2] Barberio, C., Withers, A., Mishra, Y., Couraud, P.O., Romero, I.A., Weksler, B. et al. (2022) A human-derived neurovascular unit in vitro model to study the effects of cellular cross-talk and soluble factors on barrier integrity. Front. Cell. Neurosci. 16, 1065193 10.3389/fncel.2022.1065193 36545654 PMC9762047

[3] Heithoff, B.P., George, K.K., Phares, A.N., Zuidhoek, I.A., Munoz-Ballester, C. and Robel, S. (2021) Astrocytes are necessary for blood–brain barrier maintenance in the adult mouse brain. Glia 69, 436–472 10.1002/glia.23908 32955153 PMC7736206

[4] Siddharthan, V., Kim, Y.V., Liu, S. and Kim, K.S. (2007) Human astrocytes/astrocyte-conditioned medium and shear stress enhance the barrier properties of human brain microvascular endothelial cells. Brain Res. 1147, 39–50 10.1016/j.brainres.2007.02.029 17368578 PMC2691862

[5] Daneman, R., Zhou, L., Kebede, A.A. and Barres, B.A. (2010) Pericytes are required for blood–brain barrier integrity during embryogenesis. Nature 468, 562–566 10.1038/nature09513 20944625 PMC3241506

[6] Armulik, A., Genové, G., Mäe, M., Nisancioglu, M.H., Wallgard, E., Niaudet, C. et al. (2010) Pericytes regulate the blood–brain barrier. Nature 468, 557–561 10.1038/nature09522 20944627

[7] Schiera, G., Bono, E., Raffa, M.P., Gallo, A., Pitarresi, G.L., Di Liegro, I. et al. (2003) Synergistic effects of neurons and astrocytes on the differentiation of brain capillary endothelial cells in culture. J. Cell. Mol. Med. 7, 165–170 10.1111/j.1582-4934.2003.tb00215.x 12927055 PMC6740229

[8] Maoz, B.M., Herland, A., FitzGerald, E.A., Grevesse, T., Vidoudez, C., Pacheco, A.R. et al. (2018) A linked organ-on-chip model of the human neurovascular unit reveals the metabolic coupling of endothelial and neuronal cells. Nat. Biotechnol. 36, 865–874 10.1038/nbt.4226 30125269 PMC9254231

[9] Yang, T., Guo, R. and Zhang, F. (2019) Brain perivascular macrophages: recent advances and implications in health and diseases. CNS Neurosci. Ther. 25, 1318–1328 10.1111/cns.13263 31749316 PMC7154594

[10] Haruwaka, K., Ikegami, A., Tachibana, Y., Ohno, N., Konishi, H., Hashimoto, A. et al. (2019) Dual microglia effects on blood brain barrier permeability induced by systemic inflammation. Nat. Commun. 10, 5816 10.1038/s41467-019-13812-z 31862977 PMC6925219

[11] Engelhardt, B., Vajkoczy, P. and Weller, R.O. (2017) The movers and shapers in immune privilege of the CNS. Nat. Immunol. 18, 123–131 10.1038/ni.3666 28092374

[12] Abbott, N.J., Rönnbäck, L. and Hansson, E. (2006) Astrocyte–endothelial interactions at the blood–brain barrier. Nat. Rev. Neurosci. 7, 41–53 10.1038/nrn1824 16371949

[13] Igarashi, Y., Utsumi, H., Chiba, H., Yamada-Sasamori, Y., Tobioka, H., Kamimura, Y. et al. (1999) Glial cell line-derived neurotrophic factor induces barrier function of endothelial cells forming the blood–brain barrier. Biochem. Biophys. Res. Commun. 261, 108–112 10.1006/bbrc.1999.0992 10405331

[14] Lee, S.-W., Kim, W.J., Choi, Y.K., Song, H.S., Son, M.J., Gelman, I.H. et al. (2003) SSeCKS regulates angiogenesis and tight junction formation in blood–brain barrier. Nat. Med. 9, 900–906 10.1038/nm889 12808449

[15] Deli, M.A., Abrahám, C.S., Kataoka, Y. and Niwa, M. (2005) Permeability studies on in vitro blood–brain barrier models: physiology, pathology, and pharmacology. Cell. Mol. Neurobiol. 25, 59–127 10.1007/s10571-004-1377-8 15962509 PMC11529645

[16] Patabendige, A. (2012) The value of in vitro models of the blood–brain barrier and their uses. Altern. Lab. Anim. 40, 335–338 10.1177/026119291204000606 23398338

[17] Rubin, L.L. and Staddon, J.M. (1999) The cell biology of the blood–brain barrier. Annu. Rev. Neurosci. 22, 11–28 10.1146/annurev.neuro.22.1.11 10202530

[18] Patabendige, A., Skinner, R.A., Morgan, L. and Abbott, N.J. (2013) A detailed method for preparation of a functional and flexible blood–brain barrier model using porcine brain endothelial cells. Brain Res. 1521, 16–30 10.1016/j.brainres.2013.04.006 23603406 PMC3694295

[19] Hayashi, Y., Nomura, M., Yamagishi, S., Harada, S., Yamashita, J. and Yamamoto, H. (1997) Induction of various blood–brain barrier properties in non-neural endothelial cells by close apposition to co-cultured astrocytes. Glia 19, 13–26 10.1002/(SICI)1098-1136(199701)19:1<13::AID-GLIA2>3.0.CO;2-B 8989564

[20] McAllister, M.S., Krizanac-Bengez, L., Macchia, F., Naftalin, R.J., Pedley, K.C., Mayberg, M.R. et al. (2001) Mechanisms of glucose transport at the blood–brain barrier: an in vitro study. Brain Res. 904, 20–30 10.1016/S0006-8993(01)02418-0 11516408

[21] Helms, H.C., Madelung, R., Waagepetersen, H.S., Nielsen, C.U. and Brodin, B. (2012) In vitro evidence for the brain glutamate efflux hypothesis: brain endothelial cells cocultured with astrocytes display a polarized brain-to-blood transport of glutamate. Glia 60, 882–893 10.1002/glia.22321 22392649

[22] Baello, S., Iqbal, M., Gibb, W. and Matthews, S.G. (2016) Astrocyte-mediated regulation of multidrug resistance p-glycoprotein in fetal and neonatal brain endothelial cells: age-dependent effects. Physiol. Rep. 4, e12853 10.14814/phy2.12853 27796269 PMC5002904

[23] Demeuse, P., Kerkhofs, A., Struys-Ponsar, C., Knoops, B., Remacle, C. and van den Bosch de Aguilar, P. (2002) Compartmentalized coculture of rat brain endothelial cells and astrocytes: a syngenic model to study the blood–brain barrier. J. Neurosci. Methods 121, 21–31 10.1016/S0165-0270(02)00225-X 12393158

[24] Armulik, A., Genové, G. and Betsholtz, C. (2011) Pericytes: developmental, physiological, and pathological perspectives, problems, and promises. Dev. Cell 21, 193–215 10.1016/j.devcel.2011.07.001 21839917

[25] Sweeney, M.D., Ayyadurai, S. and Zlokovic, B.V. (2016) Pericytes of the neurovascular unit: key functions and signaling pathways. Nat. Neurosci. 19, 771–783 10.1038/nn.4288 27227366 PMC5745011

[26] Hill, R.A., Tong, L., Yuan, P., Murikinati, S., Gupta, S. and Grutzendler, J. (2015) Regional blood flow in the normal and ischemic brain is controlled by arteriolar smooth muscle cell contractility and not by capillary pericytes. Neuron 87, 95–110 10.1016/j.neuron.2015.06.001 26119027 PMC4487786

[27] Attwell, D., Mishra, A., Hall, C.N., O'Farrell, F.M. and Dalkara, T. (2016) What is a pericyte? J. Cereb. Blood Flow Metab. 36, 451–455 10.1177/0271678X15610340 26661200 PMC4759679

[28] Bell, R.D., Winkler, E.A., Sagare, A.P., Singh, I., LaRue, B., Deane, R. et al. (2010) Pericytes control key neurovascular functions and neuronal phenotype in the adult brain and during brain aging. Neuron 68, 409–427 10.1016/j.neuron.2010.09.043 21040844 PMC3056408

[29] Kisler, K., Nelson, A.R., Rege, S.V., Ramanathan, A., Wang, Y., Ahuja, A. et al. (2017) Pericyte degeneration leads to neurovascular uncoupling and limits oxygen supply to brain. Nat. Neurosci. 20, 406–416 10.1038/nn.4489 28135240 PMC5323291

[30] Nelson, A.R., Sagare, M.A., Wang, Y., Kisler, K., Zhao, Z. and Zlokovic, B.V. (2020) Channelrhodopsin excitation contracts brain pericytes and reduces blood flow in the aging mouse brain in vivo. Front. Aging Neurosci. 12, 108 10.3389/fnagi.2020.00108 32410982 PMC7201096

[31] Smyth, L.C.D., Rustenhoven, J., Park, T.I., Schweder, P., Jansson, D., Heppner, P.A. et al. (2018) Unique and shared inflammatory profiles of human brain endothelia and pericytes. J. Neuroinflammation 15, 138 10.1186/s12974-018-1167-8 29751771 PMC5948925

[32] Makihara, N., Arimura, K., Ago, T., Tachibana, M., Nishimura, A., Nakamura, K. et al. (2015) Involvement of platelet-derived growth factor receptor β in fibrosis through extracellular matrix protein production after ischemic stroke. Exp. Neurol. 264, 127–134 10.1016/j.expneurol.2014.12.007 25510317

[33] Montagne, A., Nation, D.A., Sagare, A.P., Barisano, G., Sweeney, M.D., Chakhoyan, A. et al. (2020) APOE4 leads to blood–brain barrier dysfunction predicting cognitive decline. Nature 581, 71–76 10.1038/s41586-020-2247-3 32376954 PMC7250000

[34] Sagare, A.P., Bell, R.D., Zhao, Z., Ma, Q., Winkler, E.A., Ramanathan, A. et al. (2013) Pericyte loss influences Alzheimer-like neurodegeneration in mice. Nat. Commun. 4, 2932 10.1038/ncomms3932 24336108 PMC3945879

[35] Ma, Q., Zhao, Z., Sagare, A.P., Wu, Y., Wang, M., Owens, N.C. et al. (2018) Blood–brain barrier-associated pericytes internalize and clear aggregated amyloid-β42 by LRP1-dependent apolipoprotein E isoform-specific mechanism. Mol. Neurodegener. 13, 57 10.1186/s13024-018-0286-0 30340601 PMC6194676

[36] Nikolakopoulou, A.M., Montagne, A., Kisler, K., Dai, Z., Wang, Y., Huuskonen, M.T. et al. (2019) Pericyte loss leads to circulatory failure and pleiotrophin depletion causing neuron loss. Nat. Neurosci. 22, 1089–1098 10.1038/s41593-019-0434-z 31235908 PMC6668719

[37] Montagne, A., Nikolakopoulou, A.M., Zhao, Z., Sagare, A.P., Si, G., Lazic, D. et al. (2018) Pericyte degeneration causes white matter dysfunction in the mouse central nervous system. Nat. Med. 24, 326–337 10.1038/nm.4482 29400711 PMC5840035

[38] Nakagomi, T., Kubo, S., Nakano-Doi, A., Sakuma, R., Lu, S., Narita, A. et al. (2015) Brain vascular pericytes following ischemia have multipotential stem cell activity to differentiate into neural and vascular lineage cells. Stem Cells 33, 1962–1974 10.1002/stem.1977 25694098

[39] Sakuma, R., Kawahara, M., Nakano-Doi, A., Takahashi, A., Tanaka, Y., Narita, A. et al. (2016) Brain pericytes serve as microglia-generating multipotent vascular stem cells following ischemic stroke. J. Neuroinflammation 13, 57 10.1186/s12974-016-0523-9 26952098 PMC4782566

[40] Virgintino, D., Girolamo, F., Errede, M., Capobianco, C., Robertson, D., Stallcup, W.B. et al. (2007) An intimate interplay between precocious, migrating pericytes and endothelial cells governs human fetal brain angiogenesis. Angiogenesis 10, 35–45 10.1007/s10456-006-9061-x 17225955

[41] Hellström, M., Gerhardt, H., Kalén, M., Li, X., Eriksson, U., Wolburg, H. et al. (2001) Lack of pericytes leads to endothelial hyperplasia and abnormal vascular morphogenesis. J. Cell Biol. 153, 543–553 10.1083/jcb.153.3.543 11331305 PMC2190573

[42] Hellström, M., Kalén, M., Lindahl, P., Abramsson, A. and Betsholtz, C. (1999) Role of PDGF-B and PDGFR-beta in recruitment of vascular smooth muscle cells and pericytes during embryonic blood vessel formation in the mouse. Development 126, 3047–3055 10.1242/dev.126.14.3047 10375497

[43] Abdullahi, W., Tripathi, D. and Ronaldson, P.T. (2018) Blood–brain barrier dysfunction in ischemic stroke: targeting tight junctions and transporters for vascular protection. Am. J. Physiol. Cell Physiol. 315, C343–c356 10.1152/ajpcell.00095.2018 29949404 PMC6171039

[44] Munji, R.N., Soung, A.L., Weiner, G.A., Sohet, F., Semple, B.D., Trivedi, A. et al. (2019) Profiling the mouse brain endothelial transcriptome in health and disease models reveals a core blood–brain barrier dysfunction module. Nat. Neurosci. 22, 1892–1902 10.1038/s41593-019-0497-x 31611708 PMC6858546

[45] Sweeney, M.D., Sagare, A.P. and Zlokovic, B.V. (2018) Blood–brain barrier breakdown in Alzheimer disease and other neurodegenerative disorders. Nat. Rev. Neurol. 14, 133–150 10.1038/nrneurol.2017.188 29377008 PMC5829048

[46] Lopes Pinheiro, M.A., Kooij, G., Mizee, M.R., Kamermans, A., Enzmann, G., Lyck, R. et al. (2016) Immune cell trafficking across the barriers of the central nervous system in multiple sclerosis and stroke. Biochim. Biophys. Acta 1862, 461–471 10.1016/j.bbadis.2015.10.018 26527183

[47] Spencer, J.I., Bell, J.S. and DeLuca, G.C. (2018) Vascular pathology in multiple sclerosis: reframing pathogenesis around the blood–brain barrier. J. Neurol. Neurosurg. Psychiatry 89, 42–52 10.1136/jnnp-2017-316011 28860328

[48] Marchi, N., Banjara, M. and Janigro, D. (2016) Blood–brain barrier, bulk flow, and interstitial clearance in epilepsy. J. Neurosci. Methods 260, 118–124 10.1016/j.jneumeth.2015.06.011 26093166 PMC4835226

[49] Dadas, A. and Janigro, D. (2019) Breakdown of blood brain barrier as a mechanism of post-traumatic epilepsy. Neurobiol. Dis. 123, 20–26 10.1016/j.nbd.2018.06.022 30030025 PMC6794150

[50] Oby, E. and Janigro, D. (2006) The blood–brain barrier and epilepsy. Epilepsia 47, 1761–1774 10.1111/j.1528-1167.2006.00817.x 17116015

[51] Patabendige, A., Michael, B.D., Craig, A.G. and Solomon, T. (2018) Brain microvascular endothelial-astrocyte cell responses following Japanese encephalitis virus infection in an in vitro human blood–brain barrier model. Mol. Cell Neurosci. 89, 60–70 10.1016/j.mcn.2018.04.002 29635016 PMC5984247

[52] Michael, B.D., Bricio-Moreno, L., Sorensen, E.W., Miyabe, Y., Lian, J., Solomon, T. et al. (2020) Astrocyte- and neuron-derived CXCL1 drives neutrophil transmigration and blood–brain barrier permeability in viral encephalitis. Cell Rep. 32, 108150 10.1016/j.celrep.2020.108150 32937134 PMC7548103

[53] Michael, B.D., Griffiths, M.J., Granerod, J., Brown, D., Keir, G., Wnęk, M. et al. (2016) The interleukin-1 balance during encephalitis is associated with clinical severity, blood–brain barrier permeability, neuroimaging changes, and disease outcome. J. Infect. Dis. 213, 1651–1660 10.1093/infdis/jiv771 26712949 PMC4837908

[54] Erickson, M.A., Rhea, E.M., Knopp, R.C. and Banks, W.A. (2021) Interactions of SARS-CoV-2 with the blood–brain barrier. Int. J. Mol. Sci. 22, 2681 10.3390/ijms22052681 33800954 PMC7961671

[55] Perrin, P., Collongues, N., Baloglu, S., Bedo, D., Bassand, X., Lavaux, T. et al. (2020) Cytokine release syndrome-associated encephalopathy in patients with COVID-19. Eur. J. Neurol. 28, 248–258 10.1111/ene.14491 32853434 PMC7461405

[56] Buzhdygan, T.P., DeOre, B.J., Baldwin-Leclair, A., Bullock, T.A., McGary, H.M., Khan, J.A. et al. (2020) The SARS-CoV-2 spike protein alters barrier function in 2D static and 3D microfluidic in-vitro models of the human blood–brain barrier. Neurobiol. Dis. 146, 105131 10.1016/j.nbd.2020.105131 33053430 PMC7547916

[57] Banks, W.A., Ercal, N. and Price, T.O. (2006) The blood–brain barrier in neuroAIDS. Curr. HIV Res. 4, 259–266 10.2174/157016206777709447 16842079

[58] Stoddart, P., Satchell, S.C. and Ramnath, R. (2022) Cerebral microvascular endothelial glycocalyx damage, its implications on the blood–brain barrier and a possible contributor to cognitive impairment. Brain Res. 1780, 147804 10.1016/j.brainres.2022.147804 35101385

[59] Combes, V., Guillemin, G.J., Chan-Ling, T., Hunt, N.H. and Grau, G.E. (2012) The crossroads of neuroinflammation in infectious diseases: endothelial cells and astrocytes. Trends Parasitol. 28, 311–319 10.1016/j.pt.2012.05.008 22727810

[60] Moxon, C.A., Alhamdi, Y., Storm, J., Toh, J.M.H., McGuinness, D., Ko, J.Y. et al. (2020) Parasite histones are toxic to brain endothelium and link blood barrier breakdown and thrombosis in cerebral malaria. Blood Adv. 4, 2851–2864 10.1182/bloodadvances.2019001258 32579667 PMC7362376

[61] Blyth, B.J., Farhavar, A., Gee, C., Hawthorn, B., He, H., Nayak, A. et al. (2009) Validation of serum markers for blood–brain barrier disruption in traumatic brain injury. J. Neurotrauma 26, 1497–1507 10.1089/neu.2008.0738 19257803 PMC2822805

[62] Aryal, R. and Patabendige, A. (2021) Blood–brain barrier disruption in atrial fibrillation: a potential contributor to the increased risk of dementia and worsening of stroke outcomes? Open Biol. 11, 200396 10.1098/rsob.200396 33878948 PMC8059575

[63] Stamatovic, S.M., Johnson, A.M., Keep, R.F. and Andjelkovic, A.V. (2016) Junctional proteins of the blood–brain barrier: new insights into function and dysfunction. Tissue Barriers 4, e1154641 10.1080/21688370.2016.1154641 27141427 PMC4836471

[64] Ayloo, S. and Gu, C. (2019) Transcytosis at the blood–brain barrier. Curr. Opin. Neurobiol. 57, 32–38 10.1016/j.conb.2018.12.014 30708291 PMC6629499

[65] Villaseñor, R., Lampe, J., Schwaninger, M. and Collin, L. (2019) Intracellular transport and regulation of transcytosis across the blood–brain barrier. Cell. Mol. Life Sci. 76, 1081–1092 10.1007/s00018-018-2982-x 30523362 PMC6513804

[66] Erickson, M.A., Wilson, M.L. and Banks, W.A. (2020) In vitro modeling of blood–brain barrier and interface functions in neuroimmune communication. Fluids Barriers CNS 17, 26 10.1186/s12987-020-00187-3 32228633 PMC7106666

[67] Shi, K., Tian, D.C., Li, Z.G., Ducruet, A.F., Lawton, M.T. and Shi, F.D. (2019) Global brain inflammation in stroke. Lancet. Neurol. 18, 1058–1066 10.1016/S1474-4422(19)30078-X 31296369

[68] Sorby-Adams, A.J., Marcoionni, A.M., Dempsey, E.R., Woenig, J.A. and Turner, R.J. (2017) The role of neurogenic inflammation in blood–brain barrier disruption and development of cerebral oedema following acute central nervous system (CNS) injury. Int. J. Mol. Sci. 18, 1788 10.3390/ijms18081788 28817088 PMC5578176

[69] Coisne, C., Lyck, R. and Engelhardt, B. (2007) Therapeutic targeting of leukocyte trafficking across the blood–brain barrier. Inflamm. Allergy Drug Targets 6, 210–222 10.2174/187152807783334328 18220956

[70] Engelhardt, B. (2006) Regulation of immune cell entry into the central nervous system. Results Probl. Cell Differ. 43, 259–280 10.1007/400_020 17068976

[71] Blamire, A.M., Anthony, D.C., Rajagopalan, B., Sibson, N.R., Perry, V.H. and Styles, P. (2000) Interleukin-1beta -induced changes in blood–brain barrier permeability, apparent diffusion coefficient, and cerebral blood volume in the rat brain: a magnetic resonance study. J. Neurosci. 20, 8153–8159 10.1523/JNEUROSCI.20-21-08153.2000 11050138 PMC6772751

[72] Marchi, N., Fan, Q.Y., Ghosh, C., Fazio, V., Bertolini, F., Betto, G. et al. (2009) Antagonism of peripheral inflammation reduces the severity of status epilepticus. Neurobiol. Dis. 33, 171–181 10.1016/j.nbd.2008.10.002 19010416 PMC3045783

[73] Marchi, N., Granata, T., Freri, E., Ciusani, E., Ragona, F., Puvenna, V. et al. (2011) Efficacy of anti-inflammatory therapy in a model of acute seizures and in a population of pediatric drug resistant epileptics. PLoS One 6, e18200 10.1371/journal.pone.0018200 21464890 PMC3065475

[74] Engelhardt, B. and Kappos, L. (2008) Natalizumab: targeting alpha4-integrins in multiple sclerosis. Neurodegener. Dis. 5, 16–22 10.1159/000109933 18075270

[75] Mahringer, A. and Fricker, G. (2016) ABC transporters at the blood–brain barrier. Expert. Opin. Drug Metab. Toxicol. 12, 499–508 10.1517/17425255.2016.1168804 26998936

[76] Gil-Martins, E., Barbosa, D.J., Silva, V., Remião, F. and Silva, R. (2020) Dysfunction of ABC transporters at the blood–brain barrier: role in neurological disorders. Pharmacol. Ther. 213, 107554 10.1016/j.pharmthera.2020.107554 32320731

[77] Shah, K. and Abbruscato, T. (2014) The role of blood–brain barrier transporters in pathophysiology and pharmacotherapy of stroke. Curr. Pharm. Des. 20, 1510–1522 10.2174/13816128113199990465 23789950

[78] Zhao, Z., Nelson, A.R., Betsholtz, C. and Zlokovic, B.V. (2015) Establishment and dysfunction of the blood–brain barrier. Cell 163, 1064–1078 10.1016/j.cell.2015.10.067 26590417 PMC4655822

[79] Cruz-Flores, S., Berge, E. and Whittle, I.R. (2012) Surgical decompression for cerebral oedema in acute ischaemic stroke. Cochrane Database Syst. Rev. 1, Cd003435 10.1002/14651858.CD003435.pub2 22258954 PMC11491187

[80] Kerenyi, L., Kardos, L., Szász, J., Szatmári, S., Bereczki, D., Hegedüs, K. et al. (2006) Factors influencing hemorrhagic transformation in ischemic stroke: a clinicopathological comparison. Eur. J. Neurol. 13, 1251–1255 10.1111/j.1468-1331.2006.01489.x 17038041

[81] Szepesi, R., Csokonay, Á., Murnyák, B., Kouhsari, M.C., Hofgárt, G., Csiba, L. et al. (2016) Haemorrhagic transformation in ischaemic stroke is more frequent than clinically suspected - a neuropathological study. J. Neurol. Sci. 368, 4–10 10.1016/j.jns.2016.06.055 27538593

[82] Balami, J.S., Chen, R.L., Grunwald, I.Q. and Buchan, A.M. (2011) Neurological complications of acute ischaemic stroke. Lancet Neurol. 10, 357–371 10.1016/S1474-4422(10)70313-6 21247806

[83] Yang, C., Hawkins, K.E., Doré, S. and Candelario-Jalil, E. (2019) Neuroinflammatory mechanisms of blood–brain barrier damage in ischemic stroke. Am. J. Physiol. Cell Physiol. 316, C135–C153 10.1152/ajpcell.00136.2018 30379577 PMC6397344

[84] Sarvari, S., Moakedi, F., Hone, E., Simpkins, J.W. and Ren, X. (2020) Mechanisms in blood–brain barrier opening and metabolism-challenged cerebrovascular ischemia with emphasis on ischemic stroke. Metab Brain Dis. 35, 851–868 10.1007/s11011-020-00573-8 32297170 PMC7988906

[85] Turner, R.J. and Sharp, F.R. (2016) Implications of MMP9 for blood brain barrier disruption and hemorrhagic transformation following ischemic stroke. Front. Cell. Neurosci. 10, 56 10.3389/fncel.2016.00056 26973468 PMC4777722

[86] Zhang, S., An, Q., Wang, T., Gao, S. and Zhou, G. (2018) Autophagy- and MMP-2/9-mediated reduction and redistribution of ZO-1 contribute to hyperglycemia-increased blood–brain barrier permeability during early reperfusion in stroke. Neuroscience 377, 126–137 10.1016/j.neuroscience.2018.02.035 29524637

[87] Zhang, Z.G., Zhang, L., Tsang, W., Goussev, A., Powers, C., Ho, K.-L. et al. (2001) Dynamic platelet accumulation at the site of the occluded middle cerebral artery and in downstream microvessels is associated with loss of microvascular integrity after embolic middle cerebral artery occlusion. Brain Res. 912, 181–194 10.1016/S0006-8993(01)02735-4 11532435

[88] Rempe, R.G., Hartz, A.M. and Bauer, B. (2016) Matrix metalloproteinases in the brain and blood–brain barrier: versatile breakers and makers. J. Cereb. Blood Flow Metab. 36, 1481–1507 10.1177/0271678X16655551 27323783 PMC5012524

[89] Rayasam, A., Hsu, M., Kijak, J.A., Kissel, L., Hernandez, G., Sandor, M. et al. (2018) Immune responses in stroke: how the immune system contributes to damage and healing after stroke and how this knowledge could be translated to better cures? Immunology 154, 363–376 10.1111/imm.12918 29494762 PMC6002204

[90] Takeshita, Y. and Ransohoff, R.M. (2012) Inflammatory cell trafficking across the blood–brain barrier: chemokine regulation and in vitro models. Immunol. Rev. 248, 228–239 10.1111/j.1600-065X.2012.01127.x 22725965 PMC3383666

[91] Villringer, K., Sanz Cuesta, B.E., Ostwaldt, A.-C., Grittner, U., Brunecker, P., Khalil, A.A. et al. (2017) DCE-MRI blood–brain barrier assessment in acute ischemic stroke. Neurology 88, 433–440 10.1212/WNL.0000000000003566 28031392

[92] Arba, F., Leigh, R., Inzitari, D., Warach, S.J., Luby, M. and Lees, K.R. (2017) Blood–brain barrier leakage increases with small vessel disease in acute ischemic stroke. Neurology 89, 2143–2150 10.1212/WNL.0000000000004677 29070665 PMC5696647

[93] Gauberti, M., Montagne, A., Marcos-Contreras, O.A., Béhot, A.L., Maubert, E. and Vivien, D. (2013) Ultra-sensitive molecular MRI of vascular cell adhesion molecule-1 reveals a dynamic inflammatory penumbra after strokes. Stroke 44, 1988–1996 10.1161/STROKEAHA.111.000544 23743972

[94] Quenault, A., Martinez de Lizarrondo, S., Etard, O., Gauberti, M., Orset, C., Haelewyn, B. et al. (2016) Molecular magnetic resonance imaging discloses endothelial activation after transient ischaemic attack. Brain 140, 146–157 10.1093/brain/aww260 28031221 PMC5226059

[95] Li, F., Wang, Y., Yu, L., Cao, S., Wang, K., Yuan, J. et al. (2015) Viral infection of the central nervous system and neuroinflammation precede blood–brain barrier disruption during Japanese encephalitis virus infection. J. Virol. 89, 5602–5614 10.1128/JVI.00143-15 25762733 PMC4442524

[96] Miner, J.J., Daniels, B.P., Shrestha, B., Proenca-Modena, J.L., Lew, E.D., Lazear, H.M. et al. (2015) The TAM receptor Mertk protects against neuroinvasive viral infection by maintaining blood–brain barrier integrity. Nat. Med. 21, 1464–1472 10.1038/nm.3974 26523970 PMC4674389

[97] Lazear, H.M., Daniels, B.P., Pinto, A.K., Huang, A.C., Vick, S.C., Doyle, S.E. et al. (2015) Interferon-λ restricts west Nile virus neuroinvasion by tightening the blood–brain barrier. Sci. Transl. Med. 7, 284ra259 10.1126/scitranslmed.aaa4304 PMC4435724 25904743

[98] Strazza, M., Pirrone, V., Wigdahl, B. and Nonnemacher, M.R. (2011) Breaking down the barrier: the effects of HIV-1 on the blood–brain barrier. Brain Res. 1399, 96–115 10.1016/j.brainres.2011.05.015 21641584 PMC3139430

[99] Persidsky, Y., Ghorpade, A., Rasmussen, J., Limoges, J., Liu, X.J., Stins, M. et al. (1999) Microglial and astrocyte chemokines regulate monocyte migration through the blood–brain barrier in human immunodeficiency virus-1 encephalitis. Am. J. Pathol. 155, 1599–1611 10.1016/S0002-9440(10)65476-4 10550317 PMC1866982

[100] Adesse, D., Gladulich, L., Alvarez-Rosa, L., Siqueira, M., Marcos, A.C., Heider, M. et al. (2022) Role of aging in blood–brain barrier dysfunction and susceptibility to SARS-CoV-2 infection: impacts on neurological symptoms of COVID-19. Fluids Barriers CNS 19, 63 10.1186/s12987-022-00357-5 35982454 PMC9386676

[101] Winter, P.M., Dung, N.M., Loan, H.T., Kneen, R., Wills, B., Thu le, T. et al. (2004) Proinflammatory cytokines and chemokines in humans with Japanese encephalitis. J. Infect. Dis. 190, 1618–1626 10.1086/423328 15478067

[102] German, A.C., Myint, K.S., Mai, N.T., Pomeroy, I., Phu, N.H., Tzartos, J. et al. (2006) A preliminary neuropathological study of Japanese encephalitis in humans and a mouse model. Trans. R. Soc. Trop. Med. Hyg. 100, 1135–1145 10.1016/j.trstmh.2006.02.008 16814333

[103] Chen, C.J., Ou, Y.C., Li, J.R., Chang, C.Y., Pan, H.C., Lai, C.Y. et al. (2014) Infection of pericytes in vitro by Japanese encephalitis virus disrupts the integrity of the endothelial barrier. J. Virol. 88, 1150–1161 10.1128/JVI.02738-13 24198423 PMC3911661

[104] Matschke, J., Lütgehetmann, M., Hagel, C., Sperhake, J.P., Schröder, A.S., Edler, C. et al. (2020) Neuropathology of patients with COVID-19 in Germany: a post-mortem case series. Lancet Neurol. 19, 919–929 10.1016/S1474-4422(20)30308-2 33031735 PMC7535629

[105] Paniz-Mondolfi, A., Bryce, C., Grimes, Z., Gordon, R.E., Reidy, J., Lednicky, J. et al. (2020) Central nervous system involvement by severe acute respiratory syndrome coronavirus-2 (SARS-CoV-2). J. Med. Virol. 92, 699–702 10.1002/jmv.25915 32314810 PMC7264598

[106] Meinhardt, J., Radke, J., Dittmayer, C., Franz, J., Thomas, C., Mothes, R. et al. (2021) Olfactory transmucosal SARS-CoV-2 invasion as a port of central nervous system entry in individuals with COVID-19. Nat. Neurosci. 24, 168–175 10.1038/s41593-020-00758-5 33257876

[107] Cho, S.M., White, N., Premraj, L., Battaglini, D., Fanning, J., Suen, J. et al. (2022) Neurological manifestations of COVID-19 in adults and children. Brain. awac332 10.1093/brain/awac332 PMC9494397 36087305

[108] Lee, M.H., Perl, D.P., Nair, G., Li, W., Maric, D., Murray, H. et al. (2021) Microvascular injury in the brains of patients with COVID-19. N. Engl. J. Med. 384, 481–483 10.1056/NEJMc2033369 33378608 PMC7787217

[109] Lee, M.H., Perl, D.P., Steiner, J., Pasternack, N., Li, W., Maric, D. et al. (2022) Neurovascular injury with complement activation and inflammation in COVID-19. Brain 145, 2555–2568 10.1093/brain/awac151 35788639 PMC9278212

[110] Bonetto, V., Pasetto, L., Lisi, I., Carbonara, M., Zangari, R., Ferrari, E. et al. (2022) Markers of blood–brain barrier disruption increase early and persistently in COVID-19 patients with neurological manifestations. Front. Immunol. 13, 1070379 10.3389/fimmu.2022.1070379 36591311 PMC9798841

[111] Hoffmann, M., Kleine-Weber, H., Schroeder, S., Krüger, N., Herrler, T., Erichsen, S. et al. (2020) SARS-CoV-2 cell entry depends on ACE2 and TMPRSS2 and is blocked by a clinically proven protease inhibitor. Cell 181, 271–280.e278 10.1016/j.cell.2020.02.052 32142651 PMC7102627

[112] Chen, R., Wang, K., Yu, J., Howard, D., French, L., Chen, Z. et al. (2020) The spatial and cell-type distribution of SARS-CoV-2 receptor ACE2 in the human and mouse brains. Front. Neurol. 11, 573095 10.3389/fneur.2020.573095 33551947 PMC7855591

[113] Wenzel, J., Lampe, J., Müller-Fielitz, H., Schuster, R., Zille, M., Müller, K. et al. (2021) The SARS-CoV-2 main protease Mpro causes microvascular brain pathology by cleaving NEMO in brain endothelial cells. Nat. Neurosci. 24, 1522–1533 10.1038/s41593-021-00926-1 34675436 PMC8553622

[114] Song, E., Zhang, C., Israelow, B., Lu-Culligan, A., Prado, A.V., Skriabine, S. et al. (2021) Neuroinvasion of SARS-CoV-2 in human and mouse brain. J. Exp. Med. 218 10.1084/jem.20202135 PMC7808299 33433624

[115] Krasemann, S., Haferkamp, U., Pfefferle, S., Woo, M.S., Heinrich, F., Schweizer, M. et al. (2022) The blood–brain barrier is dysregulated in COVID-19 and serves as a CNS entry route for SARS-CoV-2. Stem Cell Rep. 17, 307–320 10.1016/j.stemcr.2021.12.011 PMC8772030 35063125

[116] Yang, R.C., Huang, K., Zhang, H.P., Li, L., Zhang, Y.F., Tan, C. et al. (2022) SARS-CoV-2 productively infects human brain microvascular endothelial cells. J. Neuroinflammation 19, 149 10.1186/s12974-022-02514-x 35705998 PMC9198209

[117] Kim, E.S., Jeon, M.T., Kim, K.S., Lee, S., Kim, S. and Kim, D.G. (2021) Spike proteins of SARS-CoV-2 induce pathological changes in molecular delivery and metabolic function in the brain endothelial cells. Viruses 13 10.3390/v13102021 PMC8538996 34696455

[118] Zhang, L., Zhou, L., Bao, L., Liu, J., Zhu, H., Lv, Q. et al. (2021) SARS-CoV-2 crosses the blood–brain barrier accompanied with basement membrane disruption without tight junctions alteration. Signal Transduct. Target Ther. 6, 337 10.1038/s41392-021-00719-9 34489403 PMC8419672

[119] Constant, O., Barthelemy, J., Bolloré, K., Tuaillon, E., Gosselet, F., Chable-Bessia, C. et al. (2021) SARS-CoV-2 poorly replicates in cells of the human blood–brain barrier without associated deleterious effects. Front. Immunol. 12, 697329 10.3389/fimmu.2021.697329 34386007 PMC8353323

[120] Patabendige, A., Singh, A., Jenkins, S., Sen, J. and Chen, R. (2021) Astrocyte activation in neurovascular damage and repair following ischaemic stroke. Int. J. Mol. Sci. 22, 4280 10.3390/ijms22084280 33924191 PMC8074612

[121] Sánchez, K.E. and Rosenberg, G.A. (2022) Shared inflammatory pathology of stroke and COVID-19. Int. J. Mol. Sci. 23, 5150 10.3390/ijms23095150 35563537 PMC9101120

[122] Al-Ahmady, Z.S., Dickie, B.R., Aldred, I., Jasim, D.A., Barrington, J., Haley, M. et al. (2022) Selective brain entry of lipid nanoparticles in haemorrhagic stroke is linked to biphasic blood–brain barrier disruption. Theranostics 12, 4477–4497 10.7150/thno.72167 35832077 PMC9254235

[123] Merali, Z., Huang, K., Mikulis, D., Silver, F. and Kassner, A. (2017) Evolution of blood-brain-barrier permeability after acute ischemic stroke. PLoS One 12, e0171558 10.1371/journal.pone.0171558 28207745 PMC5313141

[124] Morgan, C.A., Mesquita, M., Ashioti, M., Beech, J.S., Williams, S.C.R., Irving, E. et al. (2020) Late changes in blood–brain barrier permeability in a rat tMCAO model of stroke detected by gadolinium-enhanced MRI. Neurol. Res. 42, 844–852 10.1080/01616412.2020.1786637 32600164

[125] Jiao, H., Wang, Z., Liu, Y., Wang, P. and Xue, Y. (2011) Specific role of tight junction proteins claudin-5, occludin, and ZO-1 of the blood–brain barrier in a focal cerebral ischemic insult. J. Mol. Neurosci. 44, 130–139 10.1007/s12031-011-9496-4 21318404

[126] Zhu, J., Li, Z., Ji, Z., Wu, Y., He, Y., Liu, K. et al. (2022) Glycocalyx is critical for blood–brain barrier integrity by suppressing caveolin1-dependent endothelial transcytosis following ischemic stroke. Brain Pathol. 32, e13006 10.1111/bpa.13006 34286899 PMC8713524

[127] Logsdon, A.F., Meabon, J.S., Cline, M.M., Bullock, K.M., Raskind, M.A., Peskind, E.R. et al. (2018) Blast exposure elicits blood–brain barrier disruption and repair mediated by tight junction integrity and nitric oxide dependent processes. Sci. Rep. 8, 11344 10.1038/s41598-018-29341-6 30054495 PMC6063850

[128] Hu, Y., Seker, B., Exner, C., Zhang, J., Plesnila, N. and Schwarzmaier, S.M. (2021) Longitudinal characterization of blood–brain barrier permeability after experimental traumatic brain injury by in vivo 2-photon microscopy. J. Neurotrauma 38, 399–410 10.1089/neu.2020.7271 33012249

[129] Kumar, V., Lee, J.D., Coulson, E.J. and Woodruff, T.M. (2021) A validated quantitative method for the assessment of neuroprotective barrier impairment in neurodegenerative disease models. J. Neurochem. 158, 807–817 10.1111/jnc.15119 32628780

[130] Schäfer, A., Brooke, C.B., Whitmore, A.C. and Johnston, R.E. (2011) The role of the blood–brain barrier during Venezuelan equine encephalitis virus infection. J. Virol. 85, 10682–10690 10.1128/JVI.05032-11 21849461 PMC3187510

[131] Kuroiwa, T., Ting, P., Martinez, H. and Klatzo, I. (1985) The biphasic opening of the blood–brain barrier to proteins following temporary middle cerebral artery occlusion. Acta Neuropathol. 68, 122–129 10.1007/BF00688633 3907257

[132] Huang, Z.G., Xue, D., Preston, E., Karbalai, H. and Buchan, A.M. (1999) Biphasic opening of the blood–brain barrier following transient focal ischemia: effects of hypothermia. Can. J. Neurol. Sci. 26, 298–304 10.1017/S0317167100000421 10563216

[133] Veltkamp, R., Siebing, D.A., Sun, L., Heiland, S., Bieber, K., Marti, H.H. et al. (2005) Hyperbaric oxygen reduces blood–brain barrier damage and edema after transient focal cerebral ischemia. Stroke 36, 1679–1683 10.1161/01.STR.0000173408.94728.79 16020761

[134] Durukan, A., Marinkovic, I., Strbian, D., Pitkonen, M., Pedrono, E., Soinne, L. et al. (2009) Post-ischemic blood–brain barrier leakage in rats: one-week follow-up by MRI. Brain Res. 1280, 158–165 10.1016/j.brainres.2009.05.025 19450568

[135] Strbian, D., Durukan, A., Pitkonen, M., Marinkovic, I., Tatlisumak, E., Pedrono, E. et al. (2008) The blood–brain barrier is continuously open for several weeks following transient focal cerebral ischemia. Neuroscience 153, 175–181 10.1016/j.neuroscience.2008.02.012 18367342

[136] Lin, C.Y., Chang, C., Cheung, W.M., Lin, M.H., Chen, J.J., Hsu, C.Y. et al. (2008) Dynamic changes in vascular permeability, cerebral blood volume, vascular density, and size after transient focal cerebral ischemia in rats: evaluation with contrast-enhanced magnetic resonance imaging. J. Cereb. Blood Flow Metab. 28, 1491–1501 10.1038/jcbfm.2008.42 18478021

[137] Yang, Y., Thompson, J.F., Taheri, S., Salayandia, V.M., McAvoy, T.A., Hill, J.W. et al. (2013) Early inhibition of MMP activity in ischemic rat brain promotes expression of tight junction proteins and angiogenesis during recovery. J. Cereb. Blood Flow Metab. 33, 1104–1114 10.1038/jcbfm.2013.56 23571276 PMC3705440

[138] Yang, Y., Estrada, E.Y., Thompson, J.F., Liu, W. and Rosenberg, G.A. (2007) Matrix metalloproteinase-mediated disruption of tight junction proteins in cerebral vessels is reversed by synthetic matrix metalloproteinase inhibitor in focal ischemia in rat. J. Cereb. Blood Flow Metab. 27, 697–709 10.1038/sj.jcbfm.9600375 16850029

[139] Liu, J., Jin, X., Liu, K.J. and Liu, W. (2012) Matrix metalloproteinase-2-mediated occludin degradation and caveolin-1-mediated claudin-5 redistribution contribute to blood–brain barrier damage in early ischemic stroke stage. J. Neurosci. 32, 3044–3057 10.1523/JNEUROSCI.6409-11.2012 22378877 PMC3339570

[140] Asahi, M., Wang, X., Mori, T., Sumii, T., Jung, J.C., Moskowitz, M.A. et al. (2001) Effects of matrix metalloproteinase-9 gene knock-out on the proteolysis of blood–brain barrier and white matter components after cerebral ischemia. J. Neurosci. 21, 7724–7732 10.1523/JNEUROSCI.21-19-07724.2001 11567062 PMC6762894

[141] Knowland, D., Arac, A., Sekiguchi, K.J., Hsu, M., Lutz, S.E., Perrino, J. et al. (2014) Stepwise recruitment of transcellular and paracellular pathways underlies blood–brain barrier breakdown in stroke. Neuron 82, 603–617 10.1016/j.neuron.2014.03.003 24746419 PMC4016169

[142] Krueger, M., Härtig, W., Reichenbach, A., Bechmann, I. and Michalski, D. (2013) Blood–brain barrier breakdown after embolic stroke in rats occurs without ultrastructural evidence for disrupting tight junctions. PLoS One 8, e56419 10.1371/journal.pone.0056419 23468865 PMC3582567

[143] Nahirney, P.C., Reeson, P. and Brown, C.E. (2016) Ultrastructural analysis of blood–brain barrier breakdown in the peri-infarct zone in young adult and aged mice. J. Cereb. Blood Flow Metab. 36, 413–425 10.1177/0271678X15608396 26661190 PMC4759675

[144] Zhou, M., Shi, S.X., Liu, N., Jiang, Y., Karim, M.S., Vodovoz, S.J. et al. (2021) Caveolae-mediated endothelial transcytosis across the blood–Brain barrier in acute ischemic stroke. J. Clin. Med. 10, 3795 10.3390/jcm10173795 34501242 PMC8432094

[145] Ben-Zvi, A., Lacoste, B., Kur, E., Andreone, B.J., Mayshar, Y., Yan, H. et al. (2014) Mfsd2a is critical for the formation and function of the blood–brain barrier. Nature 509, 507–511 10.1038/nature13324 24828040 PMC4134871

[146] Haley, M.J. and Lawrence, C.B. (2017) The blood–brain barrier after stroke: structural studies and the role of transcytotic vesicles. J. Cereb. Blood Flow Metab. 37, 456–470 10.1177/0271678X16629976 26823471 PMC5322831

[147] Nation, D.A., Sweeney, M.D., Montagne, A., Sagare, A.P., D'Orazio, L.M., Pachicano, M. et al. (2019) Blood–brain barrier breakdown is an early biomarker of human cognitive dysfunction. Nat. Med. 25, 270–276 10.1038/s41591-018-0297-y 30643288 PMC6367058

[148] Montagne, A., Barnes, S.R., Sweeney, M.D., Halliday, M.R., Sagare, A.P., Zhao, Z. et al. (2015) Blood–brain barrier breakdown in the aging human hippocampus. Neuron 85, 296–302 10.1016/j.neuron.2014.12.032 25611508 PMC4350773

[149] van de Haar, H.J., Burgmans, S., Jansen, J.F., van Osch, M.J., van Buchem, M.A., Muller, M. et al. (2016) Blood–brain barrier leakage in patients with early Alzheimer disease. Radiology 281, 527–535 10.1148/radiol.2016152244 27243267

[150] Ballermann, B.J., Dardik, A., Eng, E. and Liu, A. (1998) Shear stress and the endothelium. Kidney Int. Suppl. 67, S100–S108 10.1046/j.1523-1755.1998.06720.x 9736263

[151] Roux, E., Bougaran, P., Dufourcq, P. and Couffinhal, T. (2020) Fluid shear stress sensing by the endothelial layer. Front. Physiol. 11, 861 10.3389/fphys.2020.00861 32848833 PMC7396610

[152] Williams-Medina, A., Deblock, M. and Janigro, D. (2020) In vitro models of the blood–brain barrier: tools in translational medicine. Front. Med. Technol. 2, 623950 10.3389/fmedt.2020.623950 35047899 PMC8757867

[153] Ott, M.J., Olson, J.L. and Ballermann, B.J. (1995) Chronic in vitro flow promotes ultrastructural differentiation of endothelial cells. Endothelium 3, 21–30 10.3109/10623329509024655

[154] Sinha, R., Le Gac, S., Verdonschot, N., van den Berg, A., Koopman, B. and Rouwkema, J. (2016) Endothelial cell alignment as a result of anisotropic strain and flow induced shear stress combinations. Sci. Rep. 6, 29510 10.1038/srep29510 27404382 PMC4941569

[155] Vion, A.C., Perovic, T., Petit, C., Hollfinger, I., Bartels-Klein, E., Frampton, E. et al. (2020) Endothelial cell orientation and polarity are controlled by shear stress and VEGF through distinct signaling pathways. Front. Physiol. 11, 623769 10.3389/fphys.2020.623769 33737879 PMC7960671

[156] Verma, D., Bajpai, V.K., Ye, N., Maneshi, M.M., Jetta, D., Andreadis, S.T. et al. (2017) Flow induced adherens junction remodeling driven by cytoskeletal forces. Exp. Cell Res. 359, 327–336 10.1016/j.yexcr.2017.08.009 28803065

[157] Malik, J., Novakova, L., Valerianova, A., Chytilova, E., Lejsek, V., Buryskova Salajova, K. et al. (2022) Wall shear stress alteration: a local risk factor of atherosclerosis. Curr. Atheroscler. Rep. 24, 143–151 10.1007/s11883-022-00993-0 35080718

[158] Cucullo, L., Hossain, M., Puvenna, V., Marchi, N. and Janigro, D. (2011) The role of shear stress in blood–brain barrier endothelial physiology. BMC Neurosci. 12, 40 10.1186/1471-2202-12-40 21569296 PMC3103473

[159] Desai, S.Y., Marroni, M., Cucullo, L., Krizanac-Bengez, L., Mayberg, M.R., Hossain, M.T. et al. (2002) Mechanisms of endothelial survival under shear stress. Endothelium 9, 89–102 10.1080/10623320212004 12200960

[160] Santaguida, S., Janigro, D., Hossain, M., Oby, E., Rapp, E. and Cucullo, L. (2006) Side by side comparison between dynamic versus static models of blood–brain barrier in vitro: a permeability study. Brain Res. 1109, 1–13 10.1016/j.brainres.2006.06.027 16857178

[161] Desai, S.Y., McAllister, M.A., Goodrich, K., Mayberg, M.M. and Janigro, D. (2001) Gene Expression Changes and Progression to a BBB Phenotype in a Dynamic Model of the BBB. In Blood–Brain Barrier (Kobiler, D., Lustig, S. and Shapira, S., eds), Springer, Boston, MA 10.1007/978-1-4615-0579-2_6

[162] Li, Y., Zheng, J., Bird, I.M. and Magness, R.R. (2003) Effects of pulsatile shear stress on nitric oxide production and endothelial cell nitric oxide synthase expression by ovine fetoplacental artery endothelial cells. Biol. Reprod. 69, 1053–1059 10.1095/biolreprod.102.013474 12773424

[163] Kanai, A.J., Strauss, H.C., Truskey, G.A., Crews, A.L., Grunfeld, S. and Malinski, T. (1995) Shear stress induces ATP-independent transient nitric oxide release from vascular endothelial cells, measured directly with a porphyrinic microsensor. Circ. Res. 77, 284–293 10.1161/01.RES.77.2.284 7614715

[164] Sriram, K., Laughlin, J.G., Rangamani, P. and Tartakovsky, D.M. (2016) Shear-induced nitric oxide production by endothelial cells. Biophys. J. 111, 208–221 10.1016/j.bpj.2016.05.034 27410748 PMC4944664

[165] Barnes, G.D. and Piazza, G. (2022) Barriers to stroke prevention in atrial fibrillation: insights from the global anticoagulation roundtable. Int. J. Cardiol. Heart Vasc. 42, 101096 10.1016/j.ijcha.2022.101096 35942005 PMC9356154

[166] McIntyre, W.F., Diederichsen, S.Z., Freedman, B., Schnabel, R.B., Svennberg, E. and Healey, J.S. (2022) Screening for atrial fibrillation to prevent stroke: a meta-analysis. Eur. Heart J. Open 2, oeac044 10.1093/ehjopen/oeac044 35919582 PMC9305505

[167] Lippi, G., Sanchis-Gomar, F. and Cervellin, G. (2021) Global epidemiology of atrial fibrillation: an increasing epidemic and public health challenge. Int. J. Stroke 16, 217–221 10.1177/1747493019897870 31955707

[168] Wolf, P.A., Mitchell, J.B., Baker, C.S., Kannel, W.B. and D'Agostino, R.B. (1998) Impact of atrial fibrillation on mortality, stroke, and medical costs. Arch. Intern. Med. 158, 229–234 10.1001/archinte.158.3.229 9472202

[169] GBD 2019 Stroke Collaborators. (2021) Global, regional, and national burden of stroke and its risk factors, 1990–2019: a systematic analysis for the Global Burden of Disease Study 2019. Lancet Neurol. 20, 795–820 10.1016/S1474-4422(21)00252-0 34487721 PMC8443449

[170] Hindricks, G., Potpara, T., Dagres, N., Arbelo, E., Bax, J.J., Blomström-Lundqvist, C. et al. (2021) 2020 ESC guidelines for the diagnosis and management of atrial fibrillation developed in collaboration with the European Association for Cardio-Thoracic Surgery (EACTS): the task force for the diagnosis and management of atrial fibrillation of the European Society of Cardiology (ESC) developed with the special contribution of the European Heart Rhythm Association (EHRA) of the ESC. Eur. Heart J. 42, 373–498 10.1093/eurheartj/ehaa612 32860505

[171] Watson, T., Shantsila, E. and Lip, G.Y. (2009) Mechanisms of thrombogenesis in atrial fibrillation: Virchow's triad revisited. Lancet 373, 155–166 10.1016/S0140-6736(09)60040-4 19135613

[172] Galenko, O., Jacobs, V., Knight, S., Bride, D., Cutler, M.J., Muhlestein, J.B. et al. (2019) Circulating levels of biomarkers of cerebral injury in patients with atrial fibrillation. Am. J. Cardiol. 124, 1697–1700 10.1016/j.amjcard.2019.08.027 31575426

[173] Polovina, M.M., Lip, G.Y. and Potpara, T.S. (2015) Endothelial (dys)function in lone atrial fibrillation. Curr. Pharm. Des. 21, 622–645 10.2174/1381612820666140825143028 25175088

[174] Guazzi, M. and Arena, R. (2009) Endothelial dysfunction and pathophysiological correlates in atrial fibrillation. Heart 95, 102–106 10.1136/hrt.2007.135277 19109515

[175] Cai, H., Li, Z., Goette, A., Mera, F., Honeycutt, C., Feterik, K. et al. (2002) Downregulation of endocardial nitric oxide synthase expression and nitric oxide production in atrial fibrillation: potential mechanisms for atrial thrombosis and stroke. Circulation 106, 2854–2858 10.1161/01.CIR.0000039327.11661.16 12451014

[176] Yoshino, S., Yoshikawa, A., Hamasaki, S., Ishida, S., Oketani, N., Saihara, K. et al. (2013) Atrial fibrillation-induced endothelial dysfunction improves after restoration of sinus rhythm. Int. J. Cardiol. 168, 1280–1285 10.1016/j.ijcard.2012.12.006 23269316

[177] Jen, N., Yu, F., Lee, J., Wasmund, S., Dai, X., Chen, C. et al. (2013) Atrial fibrillation pacing decreases intravascular shear stress in a New Zealand white rabbit model: implications in endothelial function. Biomech. Model. Mechanobiol. 12, 735–745 10.1007/s10237-012-0437-0 22983703 PMC3548016

[178] Soldozy, S., Norat, P., Elsarrag, M., Chatrath, A., Costello, J.S., Sokolowski, J.D. et al. (2019) The biophysical role of hemodynamics in the pathogenesis of cerebral aneurysm formation and rupture. Neurosurg. Focus 47, E11 10.3171/2019.4.FOCUS19232 31261115

[179] Staarmann, B., Smith, M. and Prestigiacomo, C.J. (2019) Shear stress and aneurysms: a review. Neurosurg. Focus 47, E2 10.3171/2019.4.FOCUS19225 31261124

[180] Iadecola, C. (2017) The neurovascular unit coming of age: a journey through neurovascular coupling in health and disease. Neuron 96, 17–42 10.1016/j.neuron.2017.07.030 28957666 PMC5657612

[181] Iadecola, C. (2013) The pathobiology of vascular dementia. Neuron 80, 844–866 10.1016/j.neuron.2013.10.008 24267647 PMC3842016

[182] Krizanac-Bengez, L., Hossain, M., Fazio, V., Mayberg, M. and Janigro, D. (2006) Loss of flow induces leukocyte-mediated MMP/TIMP imbalance in dynamic in vitro blood–brain barrier model: role of pro-inflammatory cytokines. Am. J. Physiol. Cell Physiol. 291, C740–C749 10.1152/ajpcell.00516.2005 16707552

[183] Krizanac-Bengez, L., Mayberg, M.R., Cunningham, E., Hossain, M., Ponnampalam, S., Parkinson, F.E. et al. (2006) Loss of shear stress induces leukocyte-mediated cytokine release and blood–brain barrier failure in dynamic in vitro blood–brain barrier model. J. Cell Physiol. 206, 68–77 10.1002/jcp.20429 15920760

[184] Krizanac-Bengez, L., Kapural, M., Parkinson, F., Cucullo, L., Hossain, M., Mayberg, M.R. et al. (2003) Effects of transient loss of shear stress on blood–brain barrier endothelium: role of nitric oxide and IL-6. Brain Res. 977, 239–246 10.1016/S0006-8993(03)02689-1 12834884

[185] Feigin, V.L., Vos, T., Nichols, E., Owolabi, M.O., Carroll, W.M., Dichgans, M. et al. (2020) The global burden of neurological disorders: translating evidence into policy. Lancet Neurol. 19, 255–265 10.1016/S1474-4422(19)30411-9 31813850 PMC9945815

[186] Ochocinska, M.J., Zlokovic, B.V., Searson, P.C., Crowder, A.T., Kraig, R.P., Ljubimova, J.Y. et al. (2017) NIH workshop report on the trans-agency blood-brain interface workshop 2016: exploring key challenges and opportunities associated with the blood, brain and their interface. Fluids Barriers CNS 14, 12 10.1186/s12987-017-0061-6 28457227 PMC5410699

[187] Kucharz, K., Kristensen, K., Johnsen, K.B., Lund, M.A., Lønstrup, M., Moos, T. et al. (2021) Post-capillary venules are the key locus for transcytosis-mediated brain delivery of therapeutic nanoparticles. Nat. Commun. 12, 4121 10.1038/s41467-021-24323-1 34226541 PMC8257611

[188] Yao, Y., Wang, J., Liu, Y., Qu, Y., Wang, K., Zhang, Y. et al. (2022) Variants of the adeno-associated virus serotype 9 with enhanced penetration of the blood–brain barrier in rodents and primates. Nat. Biomed. Eng. 6, 1257–1271 10.1038/s41551-022-00938-7 36217021

[189] Stanton, J.A., Williams, E.I., Betterton, R.D., Davis, T.P. and Ronaldson, P.T. (2022) Targeting organic cation transporters at the blood–brain barrier to treat ischemic stroke in rats. Exp. Neurol. 357, 114181 10.1016/j.expneurol.2022.114181 35905840 PMC9620710

[190] Li, F., Zhang, Y., Li, R., Li, Y., Ding, S., Zhou, J. et al. (2023) Neuronal Serpina3n is an endogenous protector against blood brain barrier damage following cerebral ischemic stroke. J. Cereb. Blood Flow Metab 43, 241–257 10.1177/0271678X221113897 36457151 PMC9903218

[191] Achón Buil, B., Tackenberg, C. and Rust, R. (2023) Editing a gateway for cell therapy across the blood–brain barrier. Brain 146, 823–841 10.1093/brain/awac393 36397727 PMC9976985

[192] Gorick, C.M., Breza, V.R., Nowak, K.M., Cheng, V.W.T., Fisher, D.G., Debski, A.C. et al. (2022) Applications of focused ultrasound-mediated blood–brain barrier opening. Adv. Drug Deliv. Rev. 191, 114583 10.1016/j.addr.2022.114583 36272635 PMC9712235

